# Heterogeneity in Disordered Gambling: Decision-Making and Impulsivity in Gamblers Grouped by Preferred Form

**DOI:** 10.3389/fpsyt.2019.00588

**Published:** 2019-08-19

**Authors:** Steve Sharman, Luke Clark, Amanda Roberts, Rosanna Michalczuk, Rachel Cocks, Henrietta Bowden-Jones

**Affiliations:** ^1^School of Psychology, College of Applied Health and Communities, University of East London, London, United Kingdom; ^2^Department of Psychology, University of Cambridge, Cambridge, United Kingdom; ^3^Centre for Gambling Research at UBC, Department of Psychology, University of British Columbia, Vancouver, BC, Canada; ^4^School of Psychology, College of Social Science, University of Lincoln, Lincoln, United Kingdom; ^5^South London and Maudsley Trust, Bethlem Royal Hospital, London, United Kingdom; ^6^Royal Holloway, University of London, London, United Kingdom; ^7^National Problem Gambling Clinic, London, United Kingdom; ^8^Faculty of Medicine, Faculty of Medicine Centre, Imperial College London, London, United Kingdom

**Keywords:** gambling, impulsivity, decision-making, disordered gambling, heterogeneity

## Abstract

**Background:** Previous research has indicated that disordered gamblers display deficits in impulsivity and risky decision-making, compared to healthy control groups. However, disordered gamblers are not a homogenous group, and differences in performance on neurocognitive tasks may be related to the form of gambling in which an individual chooses to engage. The present study used neurocognitive tasks and questionnaire measures to ascertain group differences in gamblers grouped by preferred form of gambling.

**Method:** Treatment-seeking pathological gamblers from the National Problem Gambling Clinic, London (n = 101), completed a neurocognitive assessment comprising the Cambridge gamble task (CGT), the stop-signal task (SST), a probabilistic reversal learning task (PRL), and the Kirby Monetary Choice Questionnaire, as well as questionnaire measures of gambling severity, impulsivity, depression, and anxiety. Analyses compared gamblers who favored fixed-odds betting terminals (FOBTs) (the modal form) to gamblers who preferred other forms of gambling (non-FOBT).

**Results:** The FOBT group showed impaired decision-making under risk on the CGT compared to the non-FOBT group, choosing the likely option less on more uncertain decisions. The FOBT group made fewer perseverative errors on the PRL task, had lower depression and anxiety scores, and were less likely to have a family history of problem gambling than the non-FOBT group.

**Discussion:** Decision-making and cognitive flexibility differences between gamblers grouped by gambling type supports preferred form as an important source of heterogeneity in gambling disorder. Decision-making strategies and risk attitudes should be considered when approaching cognition-focused treatment strategies, allowing interventions to be targeted at specific cognitive deficits.

## Introduction

Pathological gambling was re-classified from an impulse control disorder to an addictive disorder in the most recent versions of the Diagnostic and Statistical Manual of Mental Disorders (DSM-5) (1) and the International Classification of Diseases and Related Health Problems (11^th^ edition) (ICD-11) (2) in acknowledgement of the parallels between behavioral and substance addictions (3). The term “disordered gambling” is used hereafter as an umbrella term for people experiencing gambling-related harm.

Disruption of executive functions has been identified as being important in the development and maintenance of addictive behaviors (4). More specifically, risky decision-making and low self-control (i.e., impulsivity) are markers that cut across different forms of addiction, through the interaction of impulsive and reflective systems for assessing reward options (5, 6). As in substance addictions, groups of pathological gamblers display statistically significant impairments in decision-making using the Iowa gambling task (IGT), selecting more cards from the disadvantageous decks (7). Pathological gamblers also show deficits in risky decision-making using the Cambridge gamble task (CGT) (8, 9), the Information Sampling Test (IST) (8), and the game of dice task (10). Brevers et al. (11) found that problem gamblers perform worse than controls on tasks assessing decision-making under both explicit risk (where the odds are known) and decisions under ambiguity (where the probabilities are unknown).

Similarly, impulsivity has been seen to be elevated in both substance addictions (12) and disordered gambling (13). Impulsivity can be measured with delay discounting tasks (i.e., impulsive choice) (14–16) as well as tests of response inhibition (i.e., impulsive action) on tasks including the stop-signal task (17), the Go–No Go task (18), and the Stroop test (19). Additionally, disordered gamblers display increased response perseveration and compulsivity on reversal learning tasks (20), although Boog et al. (21) suggest these deficits may arise as a function of reward motivation rather than cognitive inflexibility *per se*. Nevertheless, the multi-dimensional nature of impulsivity has not been fully parsed in disordered gambling. Using a thorough assessment with both neurocognitive tasks and questionnaire measures, Billieux et al. found that disordered gamblers exhibited higher urgency, lower premeditation, impairment in prepotent inhibition, and lower tolerance of delayed rewards than a control group. However, they also observed considerable heterogeneity in the impulsivity profiles of the gamblers: although disordered gamblers reported elevated impulsivity at an overall level, individual gamblers displayed atypical scores on different UPPS subscales, and the disordered gamblers were not reliably impaired across all inhibition tasks, indicating that impulsivity is not universally present in disordered gamblers (22).

In comparing problem gamblers to healthy controls, an alcohol dependent group and a Tourette syndrome group on four impulsivity-related dimensions (self-reported impulsivity, prepotent response impulsivity, choice impulsivity, and motor impulsivity), Kräplin et al., (23) found that gamblers were more impulsive than the healthy control group across all dimensions, and the problem gamblers were the only group that differed on choice impulsivity, indicating some dimensions of impulsivity although a key feature in gambling disorders, are not disorder specific (23).

Traditional models of sub-typing problem gamblers primarily rely on personality traits and clinical characteristics (24–26). Three dominant subtypes of gambler are proposed, termed “behaviorally conditioned,” “emotionally vulnerable,” and “antisocial impulsivist,” with impulsivity emphasized as a dispositional factor in the third pathway. However, approaches to subtyping gamblers to date have rarely consider the form(s) of gambling the individual engages in. The level of skill, or strategy involved in different forms of gambling, can vary: lotteries are chance games, where no single outcome is more likely than any other, whereas gambling forms such as poker offer far greater potential for experienced players to develop successful strategies (27). Studies that utilize preferred form as a source of heterogeneity commonly use a dichotomy of strategic (e.g., sports, cards) *versus* non-strategic (e.g., slots, lotteries) games, describing differences in demographic variables (28–30), personality traits (31), and gambling severity (32).

Preferred form of gambling has also been investigated preliminarily in relation to neurocognitive performance. After characterizing group deficits in pathological gamblers on the IGT and a reversal learning task, Goudriaan et al. (33) separated gamblers based on their preferred forms (slot machine gamblers and casino gamblers); the slot machine gamblers displayed greater impairments in decision-making than the casino gamblers. Using a computational model to decompose performance on the IGT, Lorains et al. (34) found that strategic gamblers were significantly influenced by both gains and losses but demonstrated an inconsistent choice style, where non-strategic gamblers were less sensitive to losses and exhibited poor learning during decision-making. Navas et al. identified non-strategic gamblers displayed higher delay discounting whereas strategic gamblers reported higher cognitive distortions and self-reported reward sensitivity (35). However, in a study by Grant et al. (36), both strategic and non-strategic gamblers were impaired compared to healthy controls on tests of cognitive flexibility or motor impulsivity, but the subgroups did not differ from each other.

In the UK, fixed-odds betting terminals (FOBTs) are a form of electronic gaming machine (EGM) located in high-street betting shops and casinos. These terminals offer multiple games with “fixed odds,” including electronic roulette as a popular form. FOBTs appear to be a particularly problematic form of gambling. Disordered gamblers are estimated to account for over 22% of money and over 25% of time spent on FOBTs in the UK (37). In a small sample of treatment-seeking pathological gamblers from the London National Problem Gambling Clinic, FOBTs were the preferred form of gambling in 60% of the sample (16). Subsequent analyses found that FOBT preference is associated with increased gambling severity (38), and that use of “gaming machines” was a significant predictor of pre-treatment dropout (39). Furthermore, in data collected from gamblers seeking residential treatment in the UK, FOBTs were the most common and fastest increasing form of gambling identified by those clients as problematic (40).

Recent meta-analyses have confirmed robust differences on neurocognitive tasks in groups with disordered gambling compared to healthy comparison groups (7, 13, 20, 41). The present study focuses specifically on disordered gamblers, by exploring heterogeneity on neurocognitive and questionnaire measures of impulsivity and risky choice. A moderately large sample of treatment-seeking pathological gamblers were grouped as a function of preferred form of gambling, distinguishing FOBTs as the modal form against a non-FOBT group comprising all other preferred forms. Considering the heterogeneity in previous studies explained by strategic *vs*. non-strategic form preferences, we predicted that FOBT preferences would also predict neurocognitive performance.

## Methods

### Participants

Treatment-seeking pathological gamblers were recruited from the National Problem Gambling Clinic, London (NPGC). Inclusion criteria were a current diagnosis of pathological gambling using the Massachusetts Gambling Screen (MAGS) (42), a 12-item gambling screen based on the DSM-IV pathological gambling criteria. This was corroborated by scores indicating problem gambling on the Problem Gambling Severity Index (PGSI > 7) (43). Exclusion criteria were the presence of neurological disorders, previous serious head injury or history of psychotic disorder, leading to exclusion of nine participants. This resulted in a final sample of 101 pathological gamblers (92 male; age M = 37.6, SD = 11.3).

The study protocol was approved by Cambridge South Research Ethics Council, Ref: 09/H0305/77. Participants gave written informed consent in accordance with the Declaration of Helsinki and were reimbursed for time and travel expenses. Participants completed a general screening questionnaire to collate demographic data including age, gender, nationality, ethnicity, education level, employment status, relationship status, and handedness. This questionnaire recorded participants’ preferred form of gambling, and family history of disordered gambling.

Participants were grouped based on their stated preferred form of gambling. The modal preferred form was FOBTs in 43 participants (age M = 36.9, SD = 11.7, 41 male). Other forms (n = 58; age M = 38.1, SD = 11; 51 male) comprised sports betting (n = 14), fruit machines (n = 13), betting on horses (n = 10), poker (n = 6), casinos (n = 6), blackjack (n = 4), online casinos (n = 2), stocks and shares (n = 2), and betting shops (n = 1). Smoking status was measured by the Fagerstrom Test for Nicotine Dependence (FTND) (44). IQ estimates were obtained from two measures, the National Adult Reading Test (NART) (45) and the composite of the Matrix Reasoning and Vocabulary tests on the Wechsler Abbreviated Scale of Intelligence (46). All participants were recruited following initial assessment at the NPGC and were either awaiting treatment (FOBT n = 27; non-FOBT n = 38), receiving psychological treatment (FOBT n = 11; non-FOBT n = 14), or had completed a course of CBT (FOBT n = 5; Non-FOBT n = 6). Groups did not differ on treatment stage distributions (χ² (2) = .33, p = .85).

### Neurocognitive Assessment

#### Kirby Monetary Choice Questionnaire (Kirby MCQ) (44)

Delay discounting was measured using the Kirby MCQ (47), a temporal discounting task involving 27 binary choices between an immediate smaller reward *versus* a larger reward available following a delay. All rewards were hypothetical monetary rewards. Larger rewards varied across three levels of magnitude (low, medium, and high). The indifference points at each magnitude are used to derive a hyperbolic k value, where higher k values indicate steeper discounting of delayed rewards and thus higher impulsivity. k Values are log transformed to reduce skew and averaged over the three magnitudes to calculate the overall discounting rate.

#### Cambridge Gamble Task (CGT) (45)

Risky decision-making was examined using the Cambridge gamble task (48). On each trial, 10 boxes are presented that are colored red or blue. The ratio of colors varies from trial to trial (9:1, 8:2, 7:3, and 6:4). The participant is instructed that a token has been hidden under one box. Each trial involves two responses. First, the participant makes a decision regarding which box color the token is hidden, and second, they place a bet of some points on their color choice. Across two conditions (in counterbalanced order), bets are offered in either an ascending or descending sequence, in fixed proportions of the current tally (5, 25, 50, 75, and 95%). Participants complete four blocks of nine trials in each of two conditions; at the start of each block the participant is endowed with 100 points. Key measures were proportion of choice of most likely outcome, deliberation time, and proportion of points bet.

#### Stop-Signal Task (SST) (46)

Response inhibition was measured using the stop-signal task (49). This is a two-choice response task, where participants are presented with a “Go” stimulus that requires a rapid response (left response key for an arrow pointing left, and right response key for an arrow pointing right). Participants were instructed to inhibit the Go response if an auditory stop signal was presented (a 300-Hz tone). These stop signals occurred on 25% of trials, a short delay after the Go stimulus. This delay was adjusted over successive stop trials using a staircase procedure, to identify a point at which the participant successfully inhibited on 50% of stop trials. The task contained five blocks of 64 trials, resulting in 80 stop trials over the task. Key measures were the median Go reaction time and the stop-signal reaction time.

#### Probabilistic Reversal Learning Task (PRL) (47)

Perseverative responding was measured with a probabilistic reversal learning task (50). This is a two-choice visual discrimination, with a red and a green stimulus randomly displayed in two of four screen locations. Selection of one stimulus is positively reinforced on 80% of trials (by the word “CORRECT” appearing on the screen); the other stimulus is incorrect (“WRONG”) on 80% of trials. After 40 trials for learning the initial discrimination, the contingencies reverse for 40 trials, such that the previously incorrect stimulus is now correct on 80% of selections. Key measures are the number of errors made in the two stages, the number of consecutive errors following the reversal (i.e., perseveration), and the number of response switches following the misleading (probabilistic) feedback.

### Self-Report Measures

Anxiety was measured using the Beck Anxiety Inventory (BAI) (51), a 21-item questionnaire measuring anxiety symptoms in the past month on a scale from 0 (not at all) to 3 (severely). Scores of less than 21 indicated low anxiety, scores of 21–35 indicate moderate anxiety, and scores of ≥36 indicated severe anxiety. Depression was measured using the Beck Depression Inventory II (BDI-II, 52), a 21-item scale with scores ranging from 0 to 3. A total BDI-II score of 0–13 indicated minimal depression, scores of 14–19 indicate mild depression, 20–28 indicate moderate depression, and scores of 29–63 indicate severe depression. Impulsivity was measured using the UPPS-S (53), a 59-item self-report scale designed to measure five subscales of impulsivity. Items are answered on a Likert scale, anchored at 1 (agree strongly) to 4 (disagree strongly). The five subscales are negative urgency, positive urgency, (lack of) planning, (lack of) perseveration, and sensation seeking. Gambling cognitions were measured using the Gambling-Related Cognitions Scale (GRCS) (54), a 23-item scale where items are presented as statements, and participants are required to respond on a Likert scale anchored at 1 (strongly disagree) and 7 (strongly agree). The GRCS can be divided in to subscales of inability to stop (five items), interpretative bias (four items), illusion of control (four items), gambling expectancies (four items), and predictive control (six items).

### Data Analysis

The neurocognitive tests that involved repeated-measures factors (Kirby MCQ: reward magnitude; CGT: color ratio and ascend/descend condition; PRL: stage) were analyzed with a mixed-factorial ANOVA with group as the between-subject factor. *Post hoc* analysis utilized *t* tests where appropriate. All data were checked for homogeneity of variance, and Greenhouse–Geisser was corrected where p > .05. Group differences on the scores on the questionnaire measures between the FOBT and non-FOBT gambling groups were analyzed using independent samples *t* tests. Chi-squared analyses were used for categorical data. Error bars represent the standard error of the mean. Data from the Kirby MCQ was log transformed prior to analysis.

## Results

The two subgroups did not differ significantly on age, gambling severity [(MAGS, (42); PGSI, (43))], IQ estimates, or nicotine dependence (**Table 1**). Although the non-FOBT group showed a trend toward having a greater proportion of females, the groups did not differ significantly on gender distribution (χ² (1) = 3.15, p = .06). The non-FOBT group (38.6%) were more likely to have a family history of problem gambling than the FOBT group (23.8%; χ²(1) = 5.21, p = .02). The FOBT group scored significantly lower than the non-FOBT group on the BDI (*t* (99) = 2.16, p = .03) and BAI (*t* (97) = 2.87, p = .005). Groups did not differ on scores on any of the UPPS-P or GRCS subscales (**Table 2**).

**Table 1:** Group differences.

Questionnaire/test		Group				Test statistics		
		FOBT (n = 43)		Non-FOBT (n = 58)		T	df	p
		Mean	*Sd*	Mean	*Sd*			
Age		36.86	*11.73*	38.1	*11*	0.55	99	0.59
FTND	44	.77	*1.86*	1.46	*2.46*	1.59	98	0.11
MAGS	42	7.19	*2.04*	7.21	*1.5*	0.06	73.4	0.96
PGSI	43	18.59	*4.5*	19.16	*4.4*	0.63	96	0.53
NART	45	115.7	*6.54*	116.13	*6.58*	0.30	92	0.76
WASI	46	103.7	*17.4*	106.3	*13.34*	0.81	72.1	0.42

**Table 2:** Questionnaire measures.

Questionnaire/test		Group			Test statistics		
	FOBT (n = 43)		Non-FOBT (n = 58)		t	df	P
	Mean	Sd	Mean	Sd			
**GRCS**							
Gambling experiences	12.79	*6.17*	13.6	*7*	.61	99	.55
Illusion of control	9.6	*5.51*	7.84	*4.89*	1.69	99	.09
Predictive control	17.67	*8.53*	15.16	*7.39*	1.59	99	.12
Inability to stop	18.98	*7.69*	18.79	*8.11*	.12	99	.91
Interpretive bias	15.26	*6.21*	15.52	*6.79*	.2	99	.84
Beck Depression Inventory	17.51	*10.1*	21.86	*9.86*	2.16	98	0.03*
Beck Anxiety Inventory	11.19	*8.88*	17.37	*11.68*	2.87	97	0.005*
**UPPS-P**							
Positive urgency	33.74	*9.17*	34.4	*9.62*	0.35	98	0.73
Negative urgency	34.91	*5.74*	36.05	*6.23*	0.94	98	0.35
Lack of perseverance	22.88	*4.74*	23.67	*5.54*	0.74	98	0.46
Lack of premeditation	26	*5.31*	26.91	*5.5*	0.83	98	0.41
Sensation seeking	34.81	*8.08*	32.04	*7.5*	1.77	98	0.08
**Kirby MCQ (ln k)**							
Magnitude—small	−3.46	*1.31*	−3.48	*1.27*	0.1	94	0.92
Magnitude—medium	−3.9	*1.27*	−3.98	*1.26*	0.32	94	0.75
Magnitude—large	−4.4	*1.5*	−4.44	*1.32*	0.16	94	0.88

Kirby MCQ: The ANOVA indicated a significant main effect of magnitude (F(1.8,173) = 52.91, p < .001), such that the k values were lower for delayed rewards of larger absolute magnitude, with significant differences between each of the three levels (lowest *t* = 5.43, all tests p < .001). The main effect of group (F(1,94) = .043, p = .84) and the magnitude x group interaction (F(1.8,173) = .051, p = .94) were not significant.

**Figure 1:**
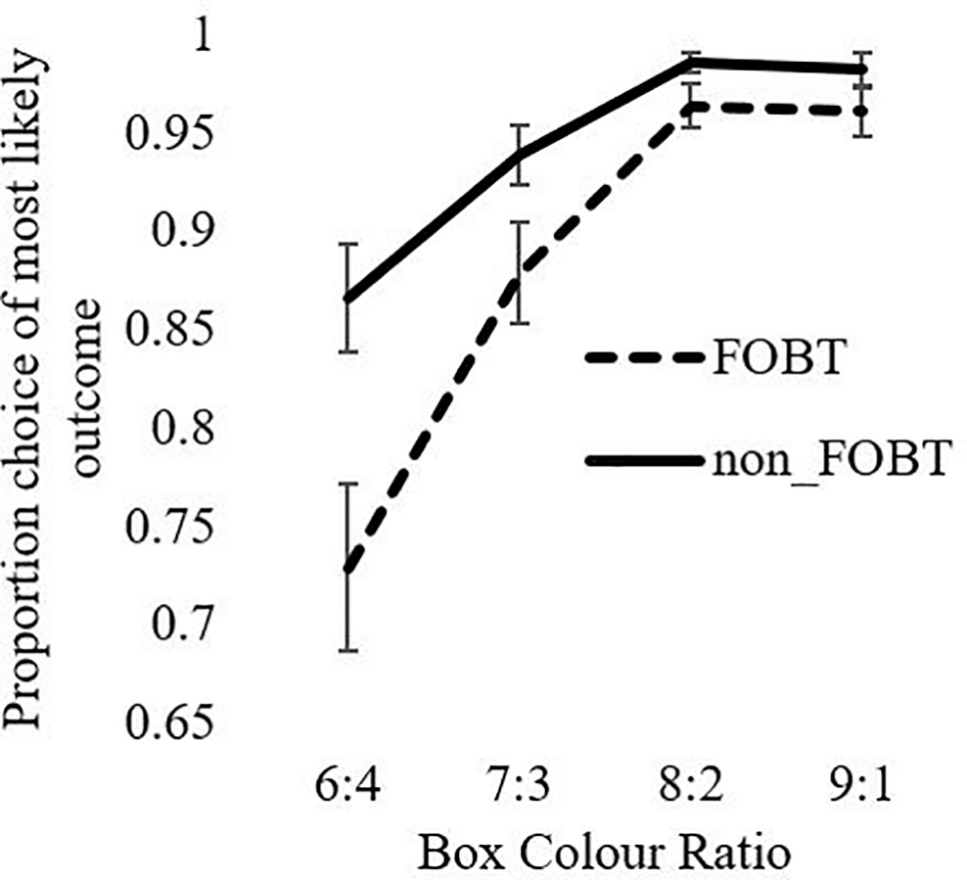
Likely outcome choice as a function of ratio.

Cambridge Gamble Task: On quality of decision-making, the ANOVA for proportion of trials on which the participant chose the more likely option showed a significant ratio x group interaction (F(1.6,121.4) = 4.78, p = .016), as well as significant main effects of ratio (F(1.6,121.4) = 43.84, p < .001) and group (F(1,76) = 9.1, p = .003). The FOBT group were less likely to choose the favorable option, and especially so at the more uncertain box ratios (6:4 ratio: *t* (56.8) = 2.84, p = .006; 7:3 *t* (52.8) = 2.13, p = .05). The 8:2 and 9:1 ratios were non-significant (lowest *t* = 1.47, p > .05), **Figure 1**.

**Figure 2:**
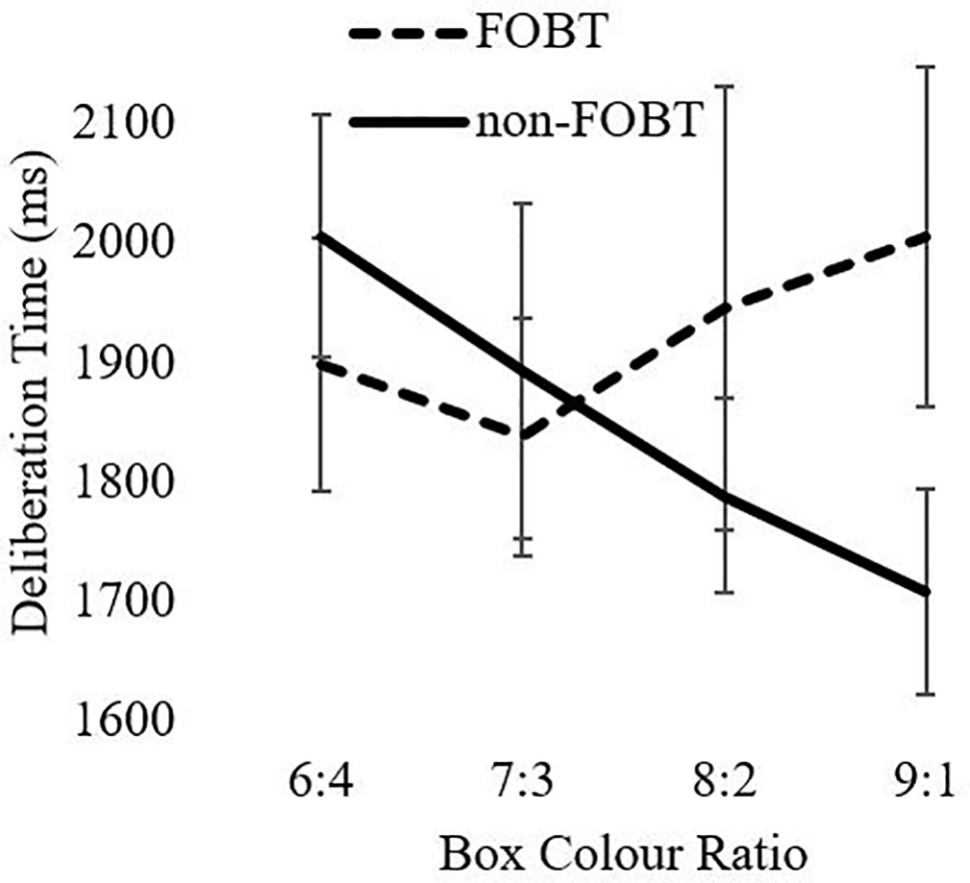
Deliberation time as a function of ratio.

An equivalent model for deliberation times indicated a significant ratio x group interaction (F(2.7,247.7) = 3.86, p = .02). The non-FOBT group demonstrated the expected pattern of longer deliberation times when the box color ratio was more evenly distributed (e.g., 6:4), than when the odds were greater (e.g., 9:1). The FOBT group demonstrated the opposite pattern, but analysis of simple effects indicated that the two groups did not differ significantly at any individual ratio (lowest *t* = .29, all p > .05). Main factors of ratio (F(2.7,247.7) = .88, p = .44) and group (F(1,93) = .243, p = .62) were not significant (**Figure 2**).

**Figure 3:**
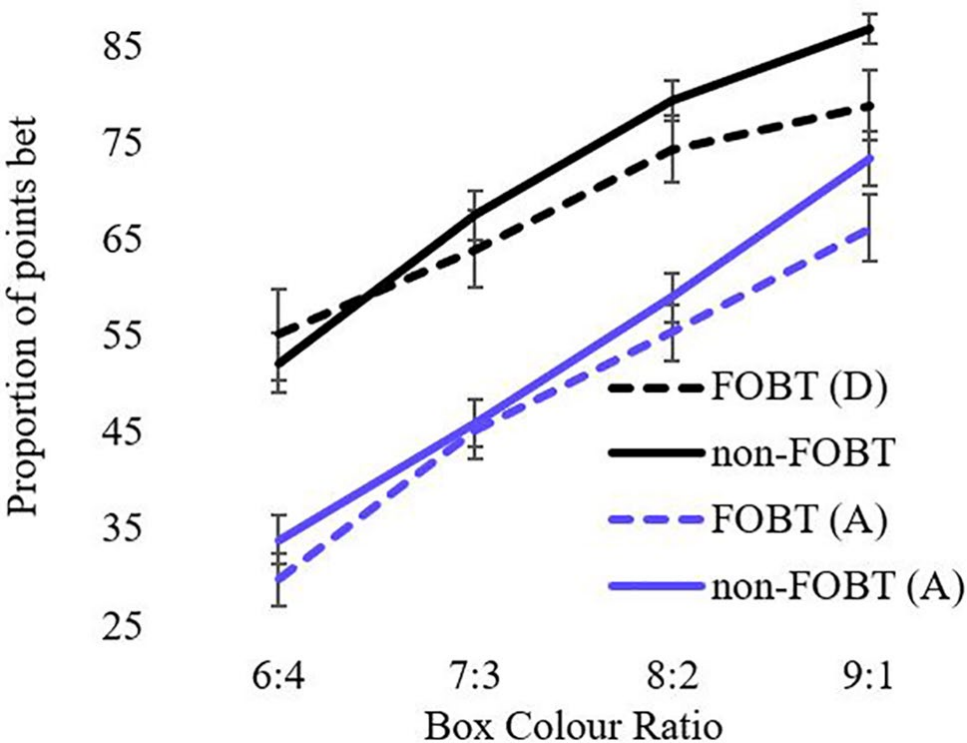
Proportion of points bet as a function of ratio.

For the analysis of betting behavior, the model shows significant main effects for ratio (F(1.6,150.3) = 256.6, p < .001) and condition (F(1,93) = 129.4, p < .001). The ratio x condition interaction was also significant (F(2.1,194) = 8.04, p < .001). Both groups bet more points in the descending condition than the ascending condition across all ratios. The main effect of group and the condition x group, ratio x group, and condition x group x ratio interactions were all non-significant (**Figure 3**). The number of “bankruptcies” (i.e., losing all points within a block, *t* (96) = .15, p = .88) and total points accrued across all trials (*t* (96) = .06, p = .95) did not differ between groups.

Stop-Signal Task: The groups did not differ on the stop-signal reaction time (FOBT M = 142.99ms, SD = 47.88; non-FOBT M = 131.86ms, SD = 41.21; *t* (80) = 1.13, p = .26). The median reaction time on “Go” trials did not differ between groups (FOBT: M = 469.93ms, SD = 113.91, non-FOBT M = 444.59ms, SD = 105.73; *t* (80) = .92, p = .36) indicating the groups did not differ in overall reaction time to go trials. In accordance with the SSD adjustment procedure, the proportion of successful stop inhibitions was close to 50% and not significantly different between groups (FOBT M = .51, SD = .06; non-FOBT M = .51, SD = .06; *t* (80) = .095, p = .92).

**Figure 4:**
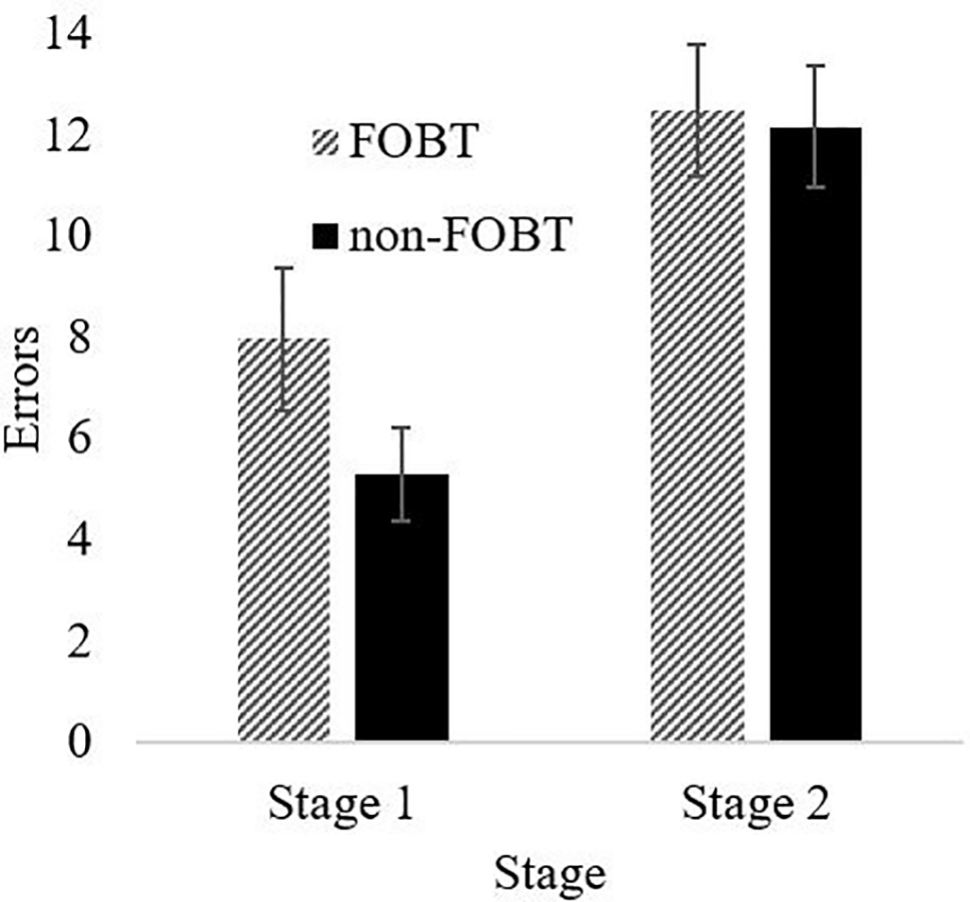
PRL errors by task stage.

**Figure 5:**
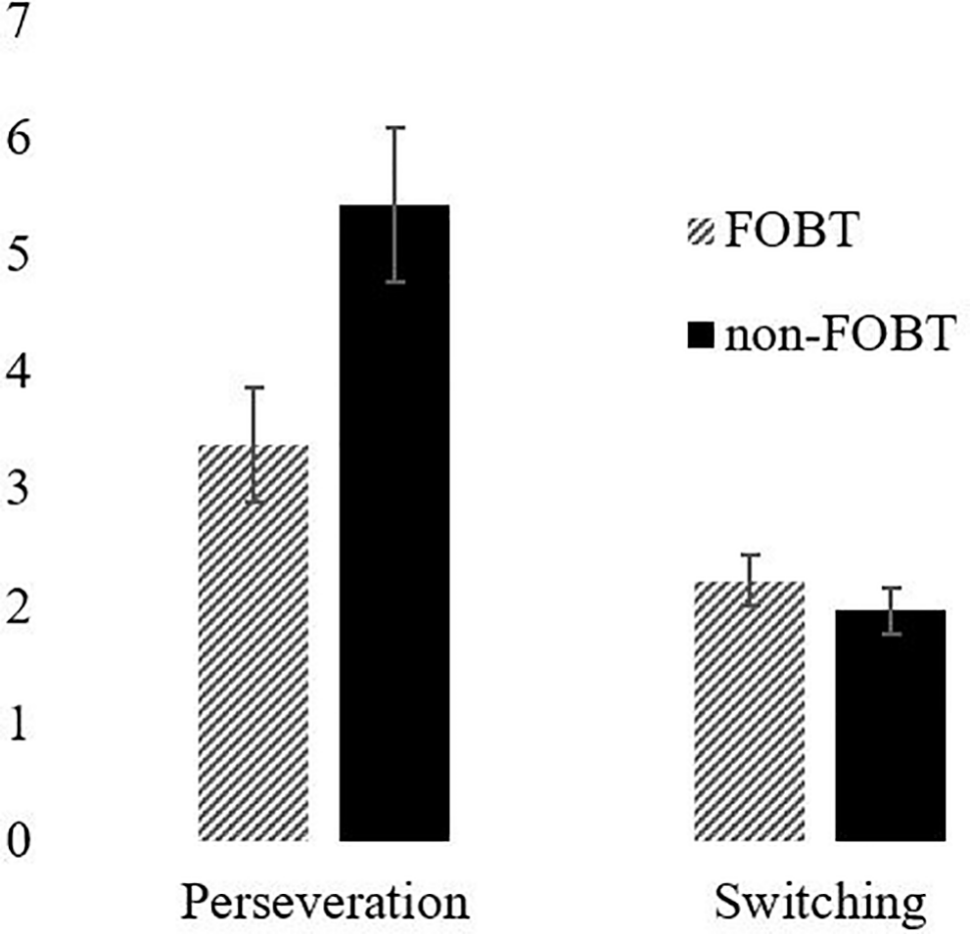
Perseveration and switching by group.

Probabilistic Reversal Learning: The ANOVA for errors by stage indicated a significant main effect of stage (F(1,87) = 36.63, p < .001), with both groups making more errors in stage 2 (**Figure 4**). The main effect of group (F(1,87) = 1.08, p = .30) and the stage x group interaction (F(1,87) = 1.57, p = .21) were non-significant. However, the groups differed significantly on perseverative errors specifically (*t* (85.9) = 2.27, p = .03); the non-FOBT group perseverated longer following the reversal switch (M = 5.43, SD = 4.8) than the FOBT group (M = 3.39, SD = 2.9) (**Figure 5**). The groups did not differ on the number of times they switched choice following misleading feedback (*t* (87) = .60, p = .55).

## Discussion

The key aim of this study was to explore the heterogeneity within a group of pathological gamblers using a psychological assessment focused on neurocognitive measures of decision-making, and questionnaire measures of impulsivity and common clinical comorbidities. Due the consistently high prevalence of FOBT gambling in UK treatment-seeking samples (including the present sample), our analyses compared FOBT gamblers against a mixed group of non-FOBT-preferring gamblers. The groups were comparable in terms of demographics and gambling severity. Analysis indicated both cognitive strengths and weaknesses in the FOBT gamblers. On the Cambridge gamble task, the FOBT group made fewer “rational” choices (i.e., of the majority color) on decisions with more uncertain odds. However, on the probabilistic reversal learning task, the FOBT group demonstrated lower levels of perseveration, potentially indicative of enhanced cognitive flexibility following the rule switch.

The CGT is a test of decision-making under risk (the odds are explicit) rather than under ambiguity. In prior research, individuals with pathological gambling differed from healthy comparison groups in terms of elevated betting and poorer quality of decision-making (8, 23). In the present study, the FOBT and non-FOBT groups did not differ in betting as a measure of impulsive and risky decision-making. However, differences were observed on decision quality, measured by the proportion of trials where the participant chooses the more likely outcome. Choice was also highly sensitive to the box ratio, with a stepwise increase in advantageous decisions as the ratios became more certain. The FOBT group made a lower proportion of advantageous choices, and this difference was strongest at the 6:4 and 7:3 ratios, where the outcomes were most uncertain. This choice of the unlikely option could be linked to the “gambler’s fallacy” (55), a classic cognitive distortion in which gamblers expect the opposite outcome to the recent sequence. On the CGT, if the token has appeared several times under the more likely color, a participant may feel that the unlikely option is “due” and opt against the rational choice. Indeed, this type of gambling distortion is prevalent in roulette, where tables often display history information regarding “hot” and “cold” numbers and colors to emphasize the recent history. FOBT players may therefore be more susceptible to gambler’s fallacy-type risky decisions.

Deliberation times to the CGT color choices also differed by preferred form, as an interaction with box ratio. The non-FOBT group showed the expected pattern whereby deliberation times became faster as the decisions became more certain (i.e., toward the 9:1 ratio). The FOBT group demonstrated the opposite pattern, with a trend toward *longer* deliberation at the more certain (9:1) color ratios. Notably, the two groups did not differ significantly at any individual box ratio. This pattern could also be explained by the conflict invoked by cognitive distortions such as the gambler’s fallacy at the most certain ratios. Anticipatory regret may be a further influence on these decisions. Regret is a powerful emotion associated with counterfactual thinking (“what might have been”) (56), and regret may increase if people do not win in a situation where they can easily imagine themselves winning (57)—for example, when choosing the majority color on the CGT. The pattern of decision latencies in the FOBT group supports the notion that probability is not the sole factor driving their color choice. This may be further expounded by gamblers who exhibit deficient emotion regulation (58).

The probabilistic reversal learning task showed that both groups made more errors in the second stage of the task, indicating increased perseveration and cognitive inflexibility. However, the results demonstrate a difference in perseveration between the two groups following the rule switch; the non-FOBT group perseverated significantly more than the FOBT group, demonstrating lower cognitive flexibility. The higher cognitive flexibility demonstrated by the FOBT group could be reflective of the cognitions associated with the different forms of gambling; the non-FOBT group contained a large number of sports and fruit machine gamblers, forms of gambling that either have relatively long outcome resolution (sports), or do not require any variation in the gambling mechanism (fruit machines), therefore do not require a great deal of quick-fire “switching” between win opportunities. Roulette on an FOBT requires the gambler to process the outcome in a number of different ways (color, odd/even, row, etc.) and then assimilate this outcome in to the decision-making process for subsequent bets, which on an FOBT can occur within 20 s. The continual updating of information requires cognitive flexibility. However, it is unclear from the current study whether a gambler with increased cognitive flexibility is drawn to FOBT machines or develops this capacity through persistent play on the terminals.

Using the Kirby Delay Discounting, both groups discounted smaller rewards more steeply than larger rewards, replicating impulsive behavior as previously demonstrated by Petry (14), Dixon et al. (15), and Michalczuk et al. (16). However, the two groups did not demonstrate any significant difference on discounting rates. The stop-signal task also failed to identify any group differences.

### Strengths and Limitations

The current study chose to focus on the heterogeneity within pathological gamblers by classifying gamblers based on their preferred form of gambling, similar to Petry (32) and Goudriaan et al. (33). Although electronic roulette and other games available on FOBTs are primarily non-strategic forms, gamblers often believe they have a strategy, or a winning formula, and will therefore often erroneously believe there are elements of skill in chance games (e.g., fruit machines) (59). This complicates the traditional strategic/non-strategic dichotomy used by Grant et al. (36) and others, as some gamblers will likely play non-strategic games in a strategic manner. In addition, the strategic/non-strategic dichotomy can be dominated by certain specific games, such as Navas et al. (35) whose “type II non-strategic gamblers” were almost exclusively slot machine gamblers. However, for the classification used in the present study, it should be noted that EGM gamblers are present in both subgroups, given that FOBTs and slot machines are both types of EGMs. These forms do differ by gambling environment: FOBTs are housed specifically in gambling facilities (bookmaker’s shops) while slot machines are also available in non-gambling venues such as pubs. The influence of these environmental factors on the cognitive differences we have observed is unclear and warrants further investigation. Furthermore, our method for categorizing gamblers was based on their stated single preferred form, but it is acknowledged that many participants also engaged in other forms of gambling.

Although the two groups did not differ on gender distribution, the sample was heavily male dominated (nine females), which prevented analyses of gender within the gambling subgroups. Our sample was treatment seeking with some variability in relation to stage of treatment (waiting list, during treatment or post-treatment). Our results may not be generalizable to the larger numbers of “at risk” gamblers. Therefore, results should be interpreted with caution. Additionally, this study did not have a non-gambling control group; differences in neurocognitive performance between gamblers and non-gamblers are well documented; the aim of this study was to better understand heterogeneity within gamblers who identify different forms as problematic.

Results indicate cognitive differences between pathological gamblers grouped by preferred form, indicating that problem gamblers are a heterogeneous group. This result should be considered when comparing gamblers as a single group to control groups, as the preferred form distribution of the gamblers could influence results.

## Ethics Statement

This study was carried out in accordance with the recommendations of Cambridge South Research Ethics Council (Ref: 09/H0305/77) with written informed consent from all subjects. All subjects gave written informed consent in accordance with the Declaration of Helsinki. The protocol was approved by the Cambridge South Research Ethics Committee.

## Author Contributions

SS was responsible for data collection, data analysis, and manuscript preparation. LC was responsible for study design, data analysis and manuscript preparation. AR was responsible for manuscript preparation. RM and RC were responsible for data collection. HB–J was responsible for study design and manuscript preparation.

## Funding

This work was supported by Medical Research Council (MRC) grants (G0802725 and G1100554, PI: LC) and completed within the University of Cambridge Behavioural and Clinical Neuroscience Institute (PI: Prof TW Robbins), supported by a consortium award from the MRC and Wellcome Trust.

## Conflict of Interest Statement

SS was funded for this work by a Cambridge Home and European Scholarship Scheme (CHESS) award. He has since been funded by GambleAware, by an internal University of Lincoln grant (Research Investment Fund), and the NIHR. He is currently funded by the Society for the Study of Addiction.

LC is the Director of the Centre for Gambling Research at UBC, which is supported by the Province of British Columbia government and the British Columbia Lottery Corporation (BCLC). The BCLC is a Canadian Crown Corporation. The Province of British Columbia government and BCLC had no involvement in the research design, methodology, conduct, analysis or write-up of the study, and impose no constraints on publishing. LC has received honoraria for serving on academic committees from the National Center for Responsible Gaming (US), and Gambling Research Exchange Ontario (Canada). He has received travel/accommodation reimbursements for speaker invitations from the National Association of Gambling Studies (Australia), the Alberta Gambling Research Institute (Canada), and the National Center for Responsible Gaming (US). He has not received any further direct or indirect payments from the gambling industry or groups substantially funded by gambling. He has received royalties from Cambridge Cognition Ltd. relating to the licensing of the Information Sampling Test; Cambridge Cognition distribute the CGT and SST reported in the present study.

AR has received funding from Santander, Public Health for Lincoln, The Royal Society, The Maurice and Jacqueline Bennett Charitable Trust, East Midlands RDS and internal University of Lincoln awards. She has no conflicts of interest.

HB–J is the Director of The National Problem Gambling Clinic which receives funds from the National Health Service and GambleAware. She is also a trustee of Action on Addiction, Patron of the Sporting Chance Clinic, Honorary Senior Lecturer, Dept of Medicine, Imperial College, Board member, International Society of Addiction Medicine, Board member of the International Society of Behavioural Addictions, Psychiatry Council member: Royal Society of Medicine, and President of the Medical Women’s Federation.

The remaining authors declare that the research was conducted in the absence of any commercial or financial relationships that could be construed as a potential conflict of interest.

## References

[1] American Psychiatric Association Diagnostic and statistical manual of mental disorders. 5th ed Arlington, VA: American Psychiatric Publishing (2013). 10.1176/appi.books.9780890425596

[2] World Health Organization (2018). International statistical classification of diseases and related health problems (11th Revision). Retrieved from https://icd.who.int/browse11/l-m/en#/http://id.who.int/icd/entity/1041487064.

[3] Grant JE Potenza MN Weinstein A Gorelick DA Introduction to behavioral addictions. Am J Drug Alcohol Abuse (2010) 36(5):233–41. 10.3109/00952990.2010.491884 PMC3164585 20560821

[4] Verdejo-Garcia A Manning V Executive functioning in gambling disorder: cognitive profiles and associations with clinical outcomes. Curr Addict Rep (2015) 2(3):214–9. 10.1007/s40429-015-0062-y

[5] Bechara A Decision making, impulse control and loss of willpower to resist drugs: a neurocognitive perspective. Nat Neurosci (2005) 8(11):1458–63. 10.1038/nn1584 16251988

[6] Verdejo-Garcia A Chong TTJ Stout JC Yücel M London ED Stages of dysfunctional decision-making in addiction. Pharmacol Biochem Behav (2017)164:99–105. 10.1016/j.pbb.2017.02.003 28216068

[7] Kovács I Richman MJ Janka Z Maraz A Andó B Decision making measured by the Iowa gambling task in alcohol use disorder and gambling disorder: a systematic review and meta-analysis. Drug Alcohol Depend (2017) 181:152–61. 10.1016/j.drugalcdep.2017.09.023 29055269

[8] Lawrence AJ Luty J Bogdan NA Sahakian BJ Clark L Problem gamblers share deficits in impulsive decision-making with alcohol-dependent individuals. Addiction (2009) 104(6):1006–15. 10.1111/j.1360-0443.2009.02533.x PMC2773538 19466924

[9] Kräplin A Dshemuchadse M Behrendt S Scherbaum S Goschke T Bühringer G Dysfunctional decision-making in pathological gambling: pattern specificity and the role of impulsivity. Psychiatry Res (2014b) 215(3):675–82. 10.1016/j.psychres.2013.12.041 24434041

[10] Brand M Kalbe E Labudda K Fujiwara E Kessler J Markowitsch HJ Decision-making impairments in patients with pathological gambling. Psychiatry Res. (2005) 133(1):91–9. 10.1016/j.psychres.2004.10.003 15698681

[11] Brevers D Cleeremans A Goudriaan AE Bechara A Kornreich C Verbanck P Decision making under ambiguity but not under risk is related to problem gambling severity. Psychiatry Res (2012) 200(2):568–74. 10.1016/j.psychres.2012.03.053 22521232

[12] Verdejo-García A Lawrence AJ Clark L Impulsivity as a vulnerability marker for substance-use disorders: review of findings from high-risk research, problem gamblers and genetic association studies. Neurosci Biobehav Rev (2008) 32(4):777–810. 10.1016/j.neubiorev.2007.11.003 18295884

[13] Chowdhury NS Livesey EJ Blaszczynski A Harris JA Pathological gambling and motor impulsivity: a systematic review with meta-analysis. J Gambl Stud (2017) 33(4):1213–39. 10.1007/s10899-017-9683-5 28255940

[14] Petry NM Pathological gamblers, with and without substance abuse disorders, discount delayed rewards at high rates. J Abnorm Psychol (2001) 110(3):482. 10.1037//0021-843X.110.3.482 11502091

[15] Dixon MR Marley J Jacobs EA Delay discounting by pathological gamblers. J Appl Behav Anal (2003) 36(4):449–58. 10.1901/jaba.2003.36-449 PMC1284461 14768665

[16] Michalczuk R Bowden-Jones H Verdejo-Garcia A Clark L Impulsivity and cognitive distortions in pathological gamblers attending the UK National Problem Gambling Clinic: a preliminary report. Psychol Med (2011) 41(12):2625–35. 10.1017/S003329171100095X PMC3206226 21733207

[17] Goudriaan AE Oosterlaan J De Beurs E Van Den Brink W Neurocognitive functions in pathological gambling: a comparison with alcohol dependence, Tourette syndrome and normal controls. Addiction (2006) 101(4):534–47. 10.1111/j.1360-0443.2006.01380.x 16548933

[18] Fuentes D Tavares H Artes R Gorenstein C Self-reported and neuropsychological measures of impulsivity in pathological gambling. J Int Neuropsychol Soc (2006) 12(06):907–12. 10.1017/S1355617706061091 17064453

[19] Kertzman S Lowengrub K Aizer A Nahum ZB Kotler M Dannon PN Stroop performance in pathological gamblers. Psychiatry Res (2006) 142(1): 1-10. 10.1016/j.psychres.2005.07.027 16626810

[20] Van Timmeren T Daams JG Van Holst RJ Goudriaan AE Compulsivity-related neurocognitive performance deficits in gambling disorder: a systematic review and meta-analysis. Neurosci Biobehav Rev (2018) 84:204–17. 10.1016/j.neubiorev.2017.11.022 29203423

[21] Boog M Höppener P Goudriaan AE Boog MC Franken IH Cognitive inflexibility in gamblers is primarily present in reward-related decision making. Front Hum Neurosci (2014) 8:569. 10.3389/fnhum.2014.00569 25165438 PMC4131672

[22] Billieux J Lagrange G Van der Linden M Lançon C Adida M Jeanningros R Investigation of impulsivity in a sample of treatment-seeking pathological gamblers: a multidimensional perspective. Psychiatry Res (2012) 198(2):291–6. 10.1016/j.psychres.2012.01.001 22421073

[23] Kräplin A Bühringer G Oosterlaan J Van Den Brink W Goschke T Goudriaan AE Dimensions and disorder specificity of impulsivity in pathological gambling. Addict Behav (2014a) 39(11):1646–51. 10.1016/j.addbeh.2014.05.021 24930455

[24] Blaszczynski A Nower L A pathways model of problem and pathological gambling. Addiction (2002) 97(5):487–99. 10.1046/j.1360-0443.2002.00015.x 12033650

[25] Ledgerwood DM Petry NM Subtyping pathological gamblers based on impulsivity, depression, and anxiety. Psychol Addict Behav (2010) 24(4):680. 10.1037/a0019906 20822191 PMC3000875

[26] Jiménez-Murcia S Granero R Stinchfield R Fernández-Aranda F Penelo E Savvidou LG Typologies of young pathological gamblers based on sociodemographic and clinical characteristics. Compr Psychiatry (2013) 54(8):1153–60. 10.1016/j.comppsych.2013.05.017 23845156

[27] Croson R Fishman P Pope DG Poker superstars: Skill or luck? Similarities between golf—thought to be a game of skill—and poker. Chance (2008) 21(4):25–8.

[28] Bonnaire C Kovess-Masfety V Guignard R Richard JB du Roscoät E Beck F Gambling type, substance abuse, health and psychosocial correlates of male and female problem gamblers in a nationally representative French sample. J Gambl Stud (2017) 33(2):343–69. 10.1007/s10899-016-9628-4 27351764

[29] Odlaug BL Marsh PJ Kim SW Grant JE Strategic vs nonstrategic gambling: characteristics of pathological gamblers based on gambling preference. Ann Clin Psychiatry (2011) 23(2):105. 21547270 PMC3179902

[30] Potenza MN Steinberg MA McLaughlin SD Wu R Rounsaville BJ O’Malley SS Gender-related differences in the characteristics of problem gamblers using a gambling helpline. Am J Psychiatr (2001) 158(9):1500–5. 10.1176/appi.ajp.158.9.1500 11532738

[31] Moragas L Granero R Stinchfield R Fernández-Aranda F Fröberg F Aymamí N Comparative analysis of distinct phenotypes in gambling disorder based on gambling preferences. BMC Psychiatry (2015) 15(1):86. 10.1186/s12888-015-0459-0 25886577 PMC4406168

[32] Petry NM A comparison of treatment-seeking pathological gamblers based on preferred gambling activity. Addiction (2003) 98(5):645–55. 10.1046/j.1360-0443.2003.00336.x 12751982

[33] Goudriaan AE Oosterlaan J de Beurs E van den Brink W Decision making in pathological gambling: a comparison between pathological gamblers, alcohol dependents, persons with Tourette syndrome, and normal controls. Cogn Brain Res (2005) 23(1):137–51. 10.1016/j.cogbrainres.2005.01.017 15795140

[34] Lorains FK Dowling NA Enticott PG Bradshaw JL Trueblood JS Stout JC Strategic and non-strategic problem gamblers differ on decision-making under risk and ambiguity. Addiction (2014) 109(7):1128–37. 10.1111/add.12494 24450756

[35] Navas JF Billieux J Perandrés-Gómez A López-Torrecillas F Cándido A Perales JC Impulsivity traits and gambling cognitions associated with gambling preferences and clinical status. International Gambling Studies (2017) 17(1):102–24. 10.1080/14459795.2016.1275739

[36] Grant JE Odlaug BL Chamberlain SR Schreiber L Neurocognitive dysfunction in strategic and non-strategic gamblers. Prog Neuropsychopharmacol Biol Psychiatry (2012) 38(2):336–40. 10.1016/j.pnpbp.2012.05.006 PMC3389298 22613186

[37] Orford J Wardle H Griffiths M What proportion of gambling is problem gambling? Estimates from the 2010 British Gambling Prevalence Survey. Int Gambl Stud (2013) 13(1):4–18.

[38] Ronzitti S Soldini E Lutri V Smith N Clerici M Bowden-Jones H Types of gambling and levels of harm: a UK study to assess severity of presentation in a treatment-seeking population. J Behav Addict (2016) 5(3):439–47. 10.1556/2006.5.2016.068 PMC5264411 27677350

[39] Ronzitti S Soldini E Smith N Bayston A Clerici M Bowden-Jones H Are treatment outcomes determined by type of gambling? A UK Study. J Gambl Stud (2018) 34(3):987–97. 10.1007/s10899-018-9752-4 29383610

[40] Sharman S Murphy R Turner JJ Roberts A Trends and patterns in UK treatment seeking gamblers: 2000–2015. Addict Behav (2019) 89:51–6. 10.1016/j.addbeh.2018.09.009 30248548

[41] MacKillop J Amlung MT Few LR Ray LA Sweet LH Munafò MR Delayed reward discounting and addictive behavior: a meta-analysis. Psychopharmacology (2011) 216(3):305–21. 10.1007/s00213-011-2229-0 PMC3201846 21373791

[42] Shaffer HJ LaBrie R Scanlan KM Cummings TN Pathological gambling among adolescents: Massachusetts gambling screen (MAGS). J Gambl Stud (1994) 10(4):339–62. 10.1007/BF02104901 24234969

[43] Ferris J Wynne H The Canadian problem gambling index. Ottawa, ON: Canadian Centre on Substance Abuse. (2001).

[44] Heatherton TF Kozlowski LT Frecker RC FAGERSTROM KO The Fagerström test for nicotine dependence: a revision of the Fagerstrom Tolerance Questionnaire. Br J Addict (1991) 86(9):1119–27. 10.1111/j.1360-0443.1991.tb01879.x 1932883

[45] Nelson HE Willison J National Adult Reading Test (NART). Windsor: Nfer-Nelson (1991).

[46] Wechsler D (1999). Wechsler abbreviated scale of intelligence. Psychological Corporation. 10.1037/t15170-000

[47] Kirby KN Petry NM Bickel WK Heroin addicts have higher discount rates for delayed rewards than non-drug-using controls. J Exp Psychol Gen (1999) 128(1):78. 10.1037//0096-3445.128.1.78 10100392

[48] Rogers RD Everitt BJ Baldacchino A Blackshaw AJ Swainson R Wynne K Dissociable deficits in the decision-making cognition of chronic amphetamine abusers, opiate abusers, patients with focal damage to prefrontal cortex, and tryptophan-depleted normal volunteers: evidence for monoaminergic mechanisms. Neuropsychopharmacology (1999) 20(4):322–39. 10.1016/S0893-133X(98)00091-8 10088133

[49] Logan GD (1994). On the ability to inhibit thought and action: a users’ guide to the stop signal paradigm.

[50] Swainson R Rogers RD Sahakian BJ Summers BA Polkey CE Robbins TW Probabilistic learning and reversal deficits in patients with Parkinson’s disease or frontal or temporal lobe lesions: possible adverse effects of dopaminergic medication. Neuropsychologia (2000) 38(5):596–612. 10.1016/S0028-3932(99)00103-7 10689037

[51] Beck AT Steer RA (1990). Manual for the Beck Anxiety Inventory.

[52] Beck AT Steer RA Brown GK (1996). Beck Depression Inventory. The psychological corporation. San Antonio, TX. 10.1037/t00742-000

[53] Cyders MA Smith GT Spillane NS Fischer S Annus AM Peterson C Integration of impulsivity and positive mood to predict risky behavior: development and validation of a measure of positive urgency. Psychol Assess (2007) 19(1):107. 10.1037/1040-3590.19.1.107 17371126

[54] Raylu N Oei TP The Gambling Related Cognitions Scale (GRCS): Development, confirmatory factor validation and psychometric properties. Addiction (2004) 99(6):757–69. 10.1111/j.1360-0443.2004.00753.x 15139874

[55] Ayton P Fischer I The hot hand fallacy and the gambler’s fallacy: two faces of subjective randomness? Mem Cognit (2004) 32(8):1369–78. 10.3758/BF03206327 15900930

[56] Kahneman D Varey CA Propensities and counterfactuals: the loser that almost won. J Pers Soc Psychol (1990) 59(6):1101. 10.1037//0022-3514.59.6.1101

[57] Holtgraves T Skeel J Cognitive biases in playing the lottery: estimating the odds and choosing the numbers. J Appl Soc Psychol (1992) 22(12):934–52. 10.1111/j.1559-1816.1992.tb00935.x

[58] Maniaci G Picone F Dimarco T Lipari A Brancato A Cannizzaro C Psychodiagnostic assessment of pathological gamblers: a focus on personality disorders, clinical syndromes and alexithymia. Int J Ment Health Addict (2015) 13(6):728–39. 10.1007/s11469-015-9550-5

[59] Griffiths MD The role of cognitive bias and skill in fruit machine gambling. Br J Psychol (1994) 85(3):351–69. 10.1111/j.2044-8295.1994.tb02529.x

